# Supplementary material for: A high-resolution mRNA expression time course of embryonic development in zebrafish
Source: eLife. 2017 Nov 16;6:e30860. doi: 10.7554/eLife.30860 (PMC5690287; doi:10.7554/eLife.30860)
Supplement: Supplementary file 6. [file elife-30860-supp6.zip › biolayout-clusters-files/Cluster129-genes.html]

Cluster129


# Cluster129: Genes

| Ensembl ID | Gene Name | Chr | Start | End | Biotype |
| --- | --- | --- | --- | --- | --- |
| ENSDARG00000007135 | cnot3a | 16 | 32156166 | 32174315 | protein\_coding |
| ENSDARG00000012261 | eftud2 | 3 | 16239206 | 16263835 | protein\_coding |
| ENSDARG00000004161 | ik | 21 | 44018321 | 44041940 | protein\_coding |
| ENSDARG00000059653 | kdm2aa | 1 | 44137403 | 44168669 | protein\_coding |
| ENSDARG00000090223 | msl1b | 12 | 26931157 | 26935657 | protein\_coding |
| ENSDARG00000054201 | rnf40 | 12 | 28795785 | 28818380 | protein\_coding |
| ENSDARG00000045146 | tomm22 | 12 | 19226664 | 19231897 | protein\_coding |
| ENSDARG00000007755 | wdr61 | 25 | 6153589 | 6165158 | protein\_coding |
| ENSDARG00000044090 | zmpste24 | 17 | 24585972 | 24594660 | protein\_coding |
| ENSDARG00000079350 | znf654 | 1 | 32915114 | 32931137 | protein\_coding |
